# Advanced Radar Absorbing Ceramic-Based Materials for Multifunctional Applications in Space Environment

**DOI:** 10.3390/ma11091730

**Published:** 2018-09-14

**Authors:** Andrea Delfini, Marta Albano, Antonio Vricella, Fabio Santoni, Giulio Rubini, Roberto Pastore, Mario Marchetti

**Affiliations:** 1 Electric and Energy Engineering (DIAEE), Department of Astronautics, Sapienza University of Rome, 00138 Rome, Italy; andrea.delfini@uniroma1.it (A.D.); antonio.vricella@uniroma1.it (A.V.); fabio.santoni@uniroma1.it (F.S.); giulio_rubini@fastwebnet.it (G.R.); 2 Italian Space Agency (ASI), 00133 Rome, Italy; marta.albano@est.asi.it; 3 Department of Mechanical and Aerospace Engineering (DIMA), Sapienza University of Rome, 00184 Rome, Italy; roberto.pastore@uniroma1.it

**Keywords:** composite materials, space environment, carbon/carbon, carbon nanotubes, thermal protection, microwave shielding, radar absorbing

## Abstract

In this review, some results of the experimental activity carried out by the authors on advanced composite materials for space applications are reported. Composites are widely employed in the aerospace industry thanks to their lightweight and advanced thermo-mechanical and electrical properties. A critical issue to tackle using engineered materials for space activities is providing two or more specific functionalities by means of single items/components. In this scenario, carbon-based composites are believed to be ideal candidates for the forthcoming development of aerospace research and space missions, since a widespread variety of multi-functional structures are allowed by employing these materials. The research results described here suggest that hybrid ceramic/polymeric structures could be employed as spacecraft-specific subsystems in order to ensure extreme temperature withstanding and electromagnetic shielding behavior simultaneously. The morphological and thermo-mechanical analysis of carbon/carbon (C/C) three-dimensional (3D) shell prototypes is reported; then, the microwave characterization of multilayered carbon-filled micro-/nano-composite panels is described. Finally, the possibility of combining the C/C bulk with a carbon-reinforced skin in a synergic arrangement is discussed, with the aid of numerical and experimental analyses.

## 1. Introduction

In recent years, the development of the aerospace industry field has moved from space environment study and exploration towards space commercialization. Exploiting space for commercial purposes entails a deep knowledge of the environment where most of the activities will be held. In particular, space mission requirements highly affect spacecraft design and structural materials selection, having to face hazards such as ionizing and ultraviolet (UV) radiation, ultra-high vacuum, plasma, atomic oxygen, micrometeoroids and debris, as well as severe temperature cycles.

Composites have long attracted the interest of materials science applied research and engineering, thanks to the capability to improve the thermo-mechanical properties of structures by lowering their mass and volume at the same time. The lightweight requirement is mandatory for military and space employments: in these fields, in fact, there is the continuing need to optimize the aerodynamic profile of the aircrafts, as well as to lower the launch and launcher expenses and to comply with the payload weight strict restrictions. In this way, polymeric components are broadly employed in spacecraft systems and subsystems such as structural elements, thermal blankets, control and conformal coatings, adhesives, lubricants, etc.

A critical issue to tackle when engineered materials are used for space activities is providing two or more specific functionalities by means of single items/components. The versatility of polymeric composites could represent a key factor to exploit in order to design, realize and validate multi-functional structures for employment in space applications. Nevertheless, introducing polymeric materials within the space environment gives rise to a number of damaging effects due to chemical structure modifications, thus degrading their thermo-mechanical properties and affecting their optical and electrical behavior. The worst effect is typically related to the surface erosion, which originates from UV and atomic oxygen irradiation during severe orbital thermal cycles.

Moreover, when the space vehicle is designed to move back into Earth’s atmosphere, the materials have to deal with the dangerous re-entry phase, when the formation of aggressive plasma occurs around the outer structure. The main issue to tackle for high velocity space vehicles, in fact, is the thermal protection from the drawback related to the critical aero-dynamic conditions. The spacecraft structures stability is hardly tested by fast chemical reactions able to cause materials sublimation and ablation. The main function of a thermal protection system (TPS) is to preserve the structure from high temperatures and oxidation phenomena generated by the interaction of the structure with the plasma environment. Different methods have been studied in order to avoid the effects of the thermal gradient occurring within the spacecraft structures; this problem is really difficult to solve because the compliance to the lightweight requirement remains mandatory for each solution. Further, the space mission requirements have to be specifically taken into account: for instance, combustion effects are rigorously analyzed in nozzle throat design and construction [1].

Ceramic materials based on carbon are considered ideal candidates to realize effective TPS for spacecraft, thanks to their low density and high thermo-mechanical resistance. In particular, the composite realized by reinforcing a carbonaceous matrix with carbon fibers—i.e., the carbon/carbon (C/C)—has been widely studied and employed for such a purpose in the last decade. This advanced material provides excellent resistance to oxidation in extreme temperature conditions, as well as good thermal conductivity, frictional properties and excellent fracture toughness [2,3,4]. Concurrent mechanisms are functional in these multi-scale carbon-based ceramics: the mechanical behavior relates to the bonding and crack propagation characteristics of the constituents, while complex transport phenomena determine the composite thermal properties. The C/C physical behavior can be tuned by the production process parameters and is dependent on the specific carbon texture selected and on the fiber architecture, as well as on the inclusion of other particulate or fibrous reinforcement. Thus, the C/C production process is conceived together with the specific item design in order to address the composite properties required to achieve the desired performances. Production of optimal C/C materials represents a really difficult task. It is worth pointing out, for example, that during the C/C fabrication process, both matrices and fibers are affected by hot treatments which induce thermo-mechanical stresses due to differential thermal expansion; such a feature is crucial in determining the final behavior of the C/C structure [5,6]. Despite a huge amount of worldwide research performed on this material, no standard production method has been established so far.

A second reason that drives aerospace researchers toward the employment of advanced engineered composites is the possibility to solve a variety of issues related to the complex interaction between space materials/structures and electromagnetic (EM) radiation. In particular, carbon-reinforced polymeric composites are believed to have a prominent role in the development of the next generation of radar absorbing materials/structures (RAM/RAS), to gain effective benefits in microwave applications, such as electromagnetic interference (EMI) mitigation and electromagnetic compatibility (EMC) solutions.

Research on EM wave absorbers was developed during World War II by Germans in response to the success the Allies were having with the early radar sets: a ferrite-based paint may be considered as the first radar absorbing material artificially conceived [7]. Later on, there was a prompt evolution of military industry research in this field toward the design and production of stealth fighters. Greater aircrafts are more easily detectable at longer distances, thus undermining the advantage of the vehicle’s high speed: for this reason, minimizing the enemy radar effectiveness became imperative as bomber size grew significantly. In most cases, stealth technology aims to reduce the radar cross section (RCS) by a suitable shape design and/or by adopting radar absorbing materials and structures [8]. RAMs are also typically exploited for a variety of applications, such as EM protection from high-intensity radiated fields (HIRF), natural phenomena (lightning), intentional EM interference (IEMI) and nuclear EM pulses (NEMP), as component of shields adopted in particle accelerators, for EM compatibility problems (equipment level shielding, anechoic chambers testing), as well as for human exposure mitigation [9,10,11,12,13,14,15,16,17].

At present, the suppression of satellite radar signatures is a strategic task for cloaking their location to enemy detection systems [18]; a stealth satellite has already been conceived for potential employment as low observable spacecraft [19]. The research on RAMs should also relate to the final product commercial applications, taking the material price into account. Large RAM panels are employed not only for military operations, but also in the field of civil engineering as well as for aerospace applications: a simple example of utility is given by satellite low reflectivity thermal insulating blankets [20].

Composite-based multi-layered structures represent a key route to achieve effective graded RAM by adding EM conductive fillers to the bulk matrix. A bi-layer absorbing structure realized by enriching porous graphite with Fe nanopowder was proposed [21]: such material was demonstrated to provide a significant microwave response in a 7 GHz bandwidth, having absorption of 43 dB at 10 GHz and lowering the losses for EM reflection beyond −20 dB. A double-layer microwave absorber was achieved by using carbonyl iron and barium ferrite powder: it showed reflection losses below −13 dB within the 6–18 GHz band and below −8 dB within the 2–18 GHz broad band [22]. A number of studies focused on the issue of how to improve the microwave absorption in broad bands by using novel materials and structures present in the scientific literature [23,24,25,26,27,28].

The nature of the filling particles is crucial for obtaining the best absorbing effectiveness. The final electromagnetic properties of the composite, in fact, are dictated by the filler EM behavior, which may exhibit a variety of electrical transport characteristics. Carbon-based composites are usually filled with graphitic micro-/nano-metric powders, which allow us to obtain a significant microwave absorption along the thicknesses in the millimeter range. For instance, the coefficient of reflection of a nickel–zinc slab with a thickness of 5 mm was measured and found to be around −11 dB at 1 GHz [29]; lately, 5.5 mm thick composite panels with carbon inclusions showed −20 dB of reflection coefficient within the 9–18 GHz band [30].

The solutions based on composites made of a polymeric matrix reinforced by carbon nanoparticles are widely analyzed. Composite materials enriched by multi-wall carbon nanotubes (MWCNT) and/or carbon nanofibers (CNF) proved to be effective EM absorbers within the microwave bands [14,26,31,32,33]. CNTs and CNFs are the thinnest carbon nano-filament existing in nature, and have long been considered for their intriguing physical behavior. In particular, as far as electrical transport is concerned, an intrinsic resistivity of 5 × 10^−5^ Ω cm (a value close to that of graphite) was measured for highly graphitic vapor-grown carbon nanofibers at room temperature [34]. Due to their excellent electrical conductivity coupled to a huge aspect ratio, relatively low weight percentages of MWCNT and CNF inclusions are able to highly affect the dielectric properties of the composite; similar results would be achievable only by adding much larger amounts of micro-size fillers.

As aforementioned, ideal materials and structures for space employment are able to provide two or more different functionalities successfully and simultaneously. In this context, above the thermal and mechanical problems typical of TPS structures, electromagnetic problems have to be taken into account. TPS made of ceramic materials such as C/C or carbon/silicon carbide (C/SiC) have been used for their mechanical and thermal properties, but few studies were focused on the EM properties of such materials. This could represent a technical gap, since in contexts such as defense or telecommunications engineering, a detailed understanding of the EM behavior of materials represents an essential prerequisite. The underlying idea here presented and discussed is to join the TPS behavior of C/C materials with the advanced EM shielding properties of carbon-based multilayered composites in order to obtain an individual spacecraft component able to face to the harsh thermal stresses induced by the space environment and to provide effective and tunable microwave absorption at the same time.

About the TPS, Section 2 describes the selection of the raw materials employed for the manufacturing of C/C shell prototypes conceived to be used in re-entry systems. A custom-developed chemical vapor infiltration (CVI) technique was adopted for the C/C fabrication; then, the manufactured structures were characterized to establish the production route reliability. Moreover, an enhanced thermal treatment was performed in order to stabilize the C/C structures by activating pyrolysis processes in order to improve the matrix microstructural geometry and thus the thermo-mechanical characteristics of the previously densified materials. In order to establish the validity of this approach, a full test campaign was planned: a microscopy characterization was carried out to analyze the morphological properties of the treated materials, then, mechanical compression tests and measurements of the coefficient of thermal expansion (CTE) were conducted to analyze the thermo-mechanical performance of the stabilized C/C.

As far as the EM absorbing materials are concerned, the microwave analysis of RAM multilayered panels made of micro-/nano-carbon reinforced polymeric composites is reported in Section 3. A reverberation chamber (RC) set-up was adopted for the EM characterization: this equipment is typically used for the study of microwave/matter interaction, as, for instance, in EMC certification tests [35,36]. A RC is a closed environment where a high intensity EM field can be attained and retained by minimal energy input, thanks to the very low absorption on the inner walls [37]. Proper EM characterization should be carried out by analyzing multiple field polarizations as well as different wave impinging directions: a RC system represents a very suitable set-up to address this goal, since is able to provide a totally random interaction between the material sample and the EM field. Measuring the coefficient of microwave absorption allows us to evaluate both the absorbing and radar cross section (ACS and RCS, respectively) of the samples under test [38]. A large RC was in-house developed at the Astronautics, Electrical and Energy Engineering Department (DIAAE) of Sapienza University of Rome, by modifying the San Marco Space Simulator in order to perform measurements of aerospace component ACS. Such apparatus can be tailored to simulate the space operating conditions (by setting chamber temperature and pressure, as well as infrared (IR)/UV irradiation), thus allowing a full space environment characterization of materials.

Finally, in Section 4, the novel concept to exploit the different skills of the above described materials in a synergic route is presented. The research results reported here suggest that hybrid ceramic/polymeric structures could be employed in order to ensure extreme temperature withstanding and electromagnetic shielding behavior simultaneously. Such typology of structure is devised in two different spacecraft configurations: as leading edge of a re-entry supersonic flight vehicle which aims to decrease RCS once back in Earth’s atmosphere, and as a thermal protection subsystem of a stealth cube satellite. One intriguing strategy to tackle the desired task has been picked out in the coupling between the C/C material and the carbon micro-/nano-powder reinforced composite; the main reason that the chemical affinity between the carbon matrix and carbon powder (basically made of the same element, i.e., graphitic carbon) should be a likely route to approach the typical issue of thermo-mechanical compatibility. The possibility of combining the C/C bulk with a carbon-reinforced skin in a synergic arrangement is thus discussed, with the aid of numerical and experimental analyses. The overall research is to develop a multi-scale carbon-based material, with the aim to improve the performance of conventional materials employed for space applications by providing advanced multifunctional components.

## 2. Materials and Methods

### 2.1. Carbon/Carbon Shell as a Thermal Protection System

The choice of materials for realizing a thermal protection system is driven by their thermo-mechanical properties and by their oxidation resistance. The guidelines can be summarized by:

- Thermal expansion compatibility between materials to avoid internal stresses due to the increasing temperature;
- Oxidation resistance for the plasma exposed layer;
- Great heat capacity for the core material to put down the temperature;
- Mechanical properties not degrading at high temperatures;
- High emissivity of the external layer in order to allow as much heat emission as possible;
- Low thermal conductivity of the inner layers in order to maintain a low temperature in the cold structure of the vehicle;
- Low catalycity of the plasma exposed surface in order to limit the surface heat flux: in fact, the outer structure could catalyze the exothermic recombination of disassociated species present in the hypersonic flow, thus resulting in heat flux increasing.

**Figure 1:**
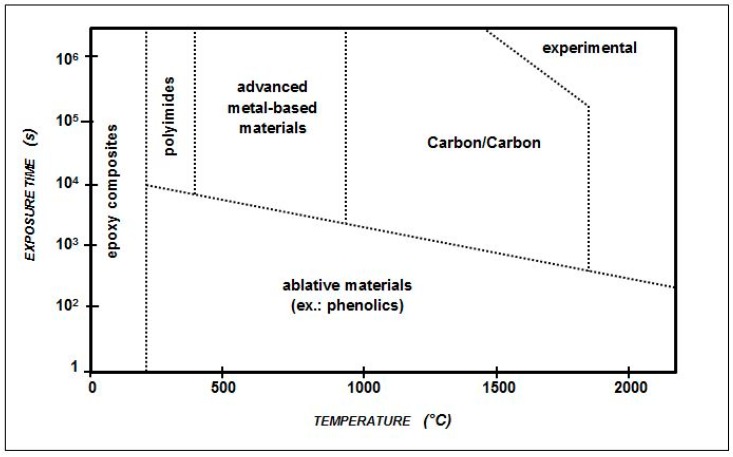
Effects of time and temperature on the ability of various composites to retain their properties [39].

Carbon-based ceramics are widely studied to obtain effective TPS for spacecraft, due to their low density and thermo-mechanical resistance. The C/C composite materials are realized by reinforcing a carbon matrix with carbon fibers and have been successfully employed to this aim in the past. C/C provides significant resistance to oxidation in aggressive environments, as well as high thermal conductivity, fracture toughness and frictional properties. Further, the C/C behavior may be addressed by tuning the fabrication parameters, as, for instance, the selection of a specific carbon fiber fabric. Thus, the C/C production process is conceived along with the specific item design, in order to address the final composite properties to provide the required performances. In Figure 1 and Table 1, some hints about the optimal C/C thermo-mechanical properties are given [39].

In general, it has been found that in carbon/carbon composites with fiber volumes below 35%, the matrix would fail between fabric layers or fiber bundles, thus limiting the maximum strength of the composite. At 35% of fiber volume and above, matrix cracking is impeded by the fiber reinforcing which, in turn, picks up the load and allows the composite to exhibit substantially higher strengths than their lower fiber-reinforced counterparts [40].

Carbon-based ceramics provide impressive capabilities of retaining their mechanical properties when subjected to very hot environmental conditions; actually, specific C/C structures showed an increase up to 20% of their mechanical performance after high temperature treatment (around 2000 °C) in inert atmosphere. C/C materials also opened novel solutions for wear-related applications, from bearing seals and electrical brushes to brake pads for heavy duty vehicles such as military, supersonic and civilian aircrafts to trucks and railways. This is possible thanks to the synergic effects of highly stiff and thermal conductive fibers intercalated into the matrix with intrinsic tribological properties; typically, C/C materials present negligible values of both friction coefficient and wear rates in fibers in parallel/orthogonal directions (0.3–0.5/0.5–0.8, and 0.05–0.1/0.1–0.3, respectively) [41,42].

In this work, the selection of the raw materials was addressed to the manufacturing of high thickness C/C prototypes conceived to be used in re-entry systems. A customized in-house developed chemical vapor infiltration technique was adopted for the C/C fabrication, then, the manufactured structures were characterized to establish the production route reliability. In order to obtain more stable characteristics of these kind of composites, high temperature stabilization cycles are usually performed, since activating pyrolysis processes can improve the matrix microstructural geometry and thus the thermo-mechanical characteristics of the previously densified materials [43]. In this perspective, an enhanced thermal treatment was carried out at a temperature of 400 °C in order to stabilize the CVI-produced C/C material. A test campaign was performed to demonstrate this approach: a morphological analysis by optical microscopy was performed to analyze and compare the chemical–physical properties of materials before and after the treatment, then a thermo-mechanical analysis—i.e., compression tests and CTE measurements—was also conducted to assess the behavior of the stabilized C/C.

In the CVI methodology for C/C fabrication, the carbonaceous matrix is derived by hydrocarbon decomposition in a hot environment, and the resulting carbon atoms move across the substrate, i.e., the carbon fiber fabric preform to be densified. Methane is widely employed as precursor for its availability and diffusion properties, despite the relatively high temperature necessary to promote the reaction (>550 °C); a carrier gas (argon, hydrogen, helium or nitrogen) is frequently flowed to enhance the hydrocarbon diffusion. The densification effectiveness depends on the adsorption properties of the porous preform surface in relation to the hydrocarbon decomposition rate and the gas flow characteristics. In particular, a proper balance between gas diffusion and adsorption kinetics must be achieved to avoid the preform pores blocking during the infiltration: surface machining steps are often required to reduce such an intrinsic effect which prevents the correct substrate filling, thus protracting the CVI processing times.

**Figure 2:**
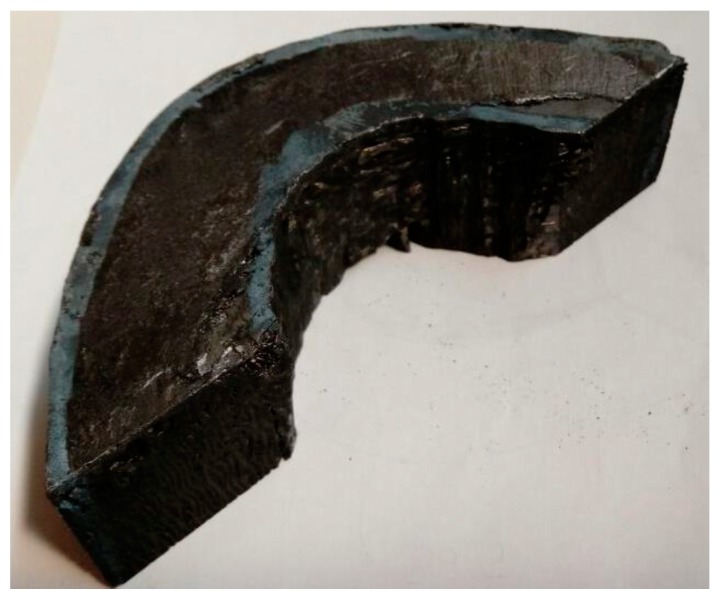
Chemical vapor infiltration (CVI)-produced C/C shell before the stabilization cycle.

The C/C shell was manufactured on the basis of the experience obtained from previous studies [44]. A 6K twill textile produced by Microtex was used in order to constitute two three-dimensional (3D) shell preforms, classified as Aldebaran and Antares. These were uniformly heated and some infiltration cycles by isothermal methane CVI treatment were applied with the aim to achieve a reasonable degree of densification (see Table 2). The as-produced shells were then refined by sandpaper grinding (see Figure 2), and some control samples were extracted from both the items before the stabilization process.

The graphitization process that thermally transforms non-graphitic carbon materials into graphite is well known in the carbon matrix composites. To be successful, several parameters must be properly conceived and tailored during the process, such as constituent composition and configuration, presence of impurities, atmosphere temperature and pressure, treatment duration, and so on [45]. The CVI process was selected as it allows us to manufacture high thermal conductivity products and because the process may result in a very pure and graphitizable carbon matrix [46]. The weak feature of the graphitization process is represented by the high temperatures of activation (about 2000–3000 °C), which may imply high processing costs.

The approach proposed in the present work was to perform a “low temperature cycle” at 400 °C (which is a sufficiently high temperature to activate the pyrolysis process) in order to remove impurities and complete the carbonization process with partial re-ordering of the matrix: such treatment is believed to increase the mechanical characteristics and stabilize the thermal properties of the final product. The shell prototypes and a series of samples for thermo-mechanical characterization were thus inserted in the furnace for the stabilization process: eight samples (four for each preform) of standard size were prepared for the compression test, four samples (two for each preform) for the CTE measurements, and four specimens (two for each preform) for the microscopy analysis. The cycle was performed at 400 °C for a period of 90 min in an argon saturated environment in order to avoid composite oxidation, activating a low vacuum system during the cycle; the heating rate was set to 10 °C/min by a PID (proportional-integral-derivative) controller, while in the cooling phase after the cycle, the samples were left within the oven for about four hours until room temperature was reached.

### 2.2. Multilayered Carbon Micro-/Nano-composite as an EM Shielding System

**Figure 3:**
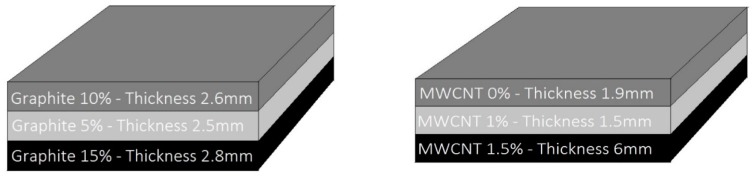
Specimens under investigation: Panel 1 (left) and Panel 2 (right) surface size is 200 mm × 200 mm.

**Figure 4:**
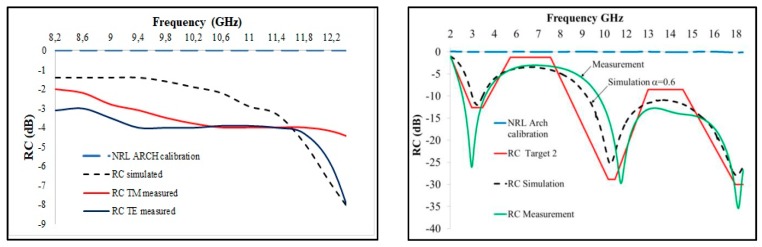
Results of reflection coefficient for Panel 1 (left) and Panel 2 (right) by free space characterization. NRL = Naval Research Lab. (RC = Reflection Coefficient; TM = Transverse Magnetic; TE = Transverse Electric).

Hereafter, the analysis of two multilayered panels made in a polymeric matrix loaded with the inclusion of different percentages of carbon-based filler is presented. The specimens under investigation are plates of epoxy resin (PRIME 20LV, purchased at Gurit, Waterville, Switzerland) loaded with different concentrations of graphite (micro-graphite powder, purchased at Sigma-Aldrich, Darmstadt, Germany) and MWCNTs (NC7000, purchased at Nanocyl, Sambreville, Belgian) by means of ultrasonication mixing methods. A sketch of the specimens is shown in Figure 3. The dimensions of both panels are 200 mm × 200 mm: the first one (Panel 1) is made of three layers of resin, with 10 wt.%, 5 wt.% and 15 wt.% of graphite inclusion, the second one (Panel 2) is made of three layers of epoxy resin, with 0 wt.%, 1 wt.% and 1.5 wt.% of MWCNT addition. The materials have been tested in previous works by analyzing their behavior at mm-waves in free space, especially within the X-band radar frequencies: good microwave absorbing capabilities were found, as reported in the plots of Figure 4 [47]. Such characterization was carried out by means of the Naval Research Lab (NRL) Arch method, i.e., in the free space set-up; the result obtained for the nano-reinforced multilayer (Panel 2) highlights the intriguing capability of this tuned composite of addressing a microwave response defined a priori.

Stealth effectiveness for employment in defense systems is the final application of RAMs. The face of air/spacecrafts can be made by nano-reinforced structures aimed at reducing vehicle detectability by surveillance radar thanks to their microwave absorbing effectiveness. It has to be stressed that it is so important to guarantee correct communications (2–4 GHz), allowing at the same time the low observability at radar frequencies (8–12 GHz).

One important issue in the evaluation of RAM characteristics is the method of analysis. Non-resonant transmission/reflection techniques are easy to perform and allow a suitable level of measurement precision over wide microwave ranges; these methods involve the measure of transmission and reflection coefficients of a material sample inserted within a waveguide or coaxial fixture. Here, the possibility of analysis within the bands L, S and C using the reverberation chamber set-up is introduced. Of particular interest for space applications is the S-band, as it is employed by weather and surface ship radar, as well as by communications satellites (for example, the satellites exploited by the National Aeronautics and Space Administration (NASA) for interconnecting to the Space Shuttle and the International Space Station).

The use of the reverberation chamber set-up is well-known and mostly adopted in electromagnetic compatibility and electromagnetic interference analysis of electronic or wireless systems [48,49,50,51,52,53]. Due to the particular electromagnetic environment, RC systems are frequently adopted to evaluate the EM absorbing capability provided by materials and structures [54,55]. The principle exploited by the RC is the chaotic distribution of the EM field, which in turns allows the materials to be hit by the EM waves from all possible directions of propagation. In this way, the limitations usually experienced by using transmission line or free-space methods in a single propagation direction are overcome. Chaotic distribution of the EM field is assured by one or more mechanical rotating paddles, also known as stirrers, which dynamically mix the electromagnetic propagation modes within the chamber [56,57]: in such a way, there is no prevalent EM field polarization within the RC.

**Figure 5:**
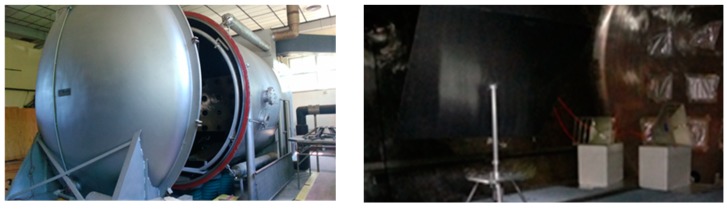
Outside (left) and inside (right) of the reverberation chamber at the Astronautics, Electrical and Energy Engineering Department (DIAEE) of the Sapienza University of Rome.

**Figure 6:**
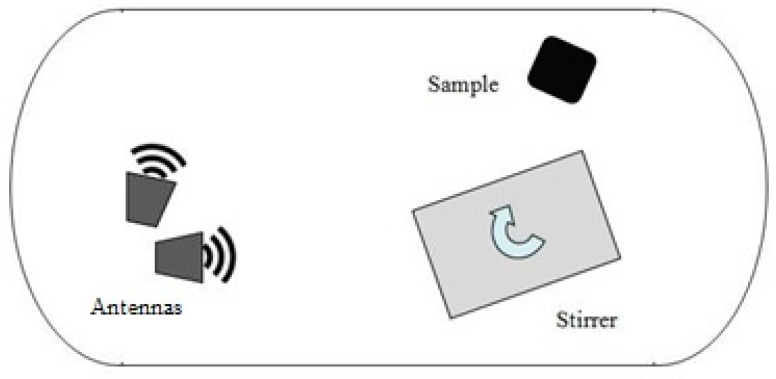
Schematic of the reverberation chamber set-up inside.

A large RC was in-house developed at the Astronautics, Electrical and Energy Engineering Department (DIAAE) of Sapienza University of Rome by modifying the San Marco Space Simulator in order to perform measurements of aerospace component ACS in the S-band (see Figure 5). Such an apparatus can be tailored to simulate the space operating conditions (by setting chamber temperature and pressure, as well as IR/UV irradiation), thus allowing a full space environment characterization of materials. The chamber has a total volume of about 40 m^3^; the fundamental EM oscillating mode has frequency of resonance around 50 MHz; thus the lowest usable operational frequency is about 250 MHz. A vector network analyzer (VNA, Anritsu model MS2026C, Anritsu, Rome, Italy) was connected to the RC to measure the EM power transmitted between two horn antennas working from 500 MHz up to 6 GHz. A Z-shaped stirrer made of copper is positioned within the RC: it is 2 m high and 1.4 m wide, and during the measurements, it is rotated in 360 independent positions with an angular resolution of 0.1°. The experimental set-up is depicted in Figure 6.

The number of the stirrer independent positions is imposed by the auto-correlation value of the measurements at each frequency against the threshold 1/e [58]. The EM intensity within the RC is calculated by averaging over the independent rotation steps: the ACS is thus evaluated in relation to the averaged quality factor (Q) of the chamber. In general, the absorbing cross section of a material sample inserted within a RC can be written as [59]:

ACS = <P_S_>/S_i_ (1)

where Ps represents the power dissipation due to the presence of the material and S_i_ = <|E_T_^2^|>/η_0_ indicates the impinging EM energy density (E_T_ being the total field magnitude and η_0_ the wave impedance of air/vacuum); the < > notation accounts for the averaging procedure over stirrer rotation and frequency stirring. Ideal conditions of field uniformity, anisotropy and un-correlation are considered in Equation (1). Due to the high modal overlapping in the RC over-mode regime, the spectral response of the chamber is strictly dependent on the perturbations due to dielectric losses. Several mechanisms of EM energy dissipation occur within the RC, as wave interaction with the chamber’s insides and antennas, as well as due to the presence of the objects under test. The contribution (Q_s_) depending on dielectric loss only is [60]:

Q_s_^−1^ = Q_l_^−1^ − Q_u_^−1^ (2)

where Q_l_ and Q_u_ are the quality factors measured for the loaded and empty RC, respectively. The dielectric loss contribution defined in Equation (2) can be also written as:

Q_s_ = ωW/<P_s_> = ωS_i_V/c<P_s_> (3)

where ω is related to the EM working frequency, W is the energy stored inside the RC, V is the chamber’s volume and c is the free space speed of light. By manipulation of Equations (2) and (3), the ACS can be expressed as:

ACS = ωV/cQ_s_ = 2πV/λ Q_s_ (4)

where λ is the EM operating wavelength. Thus, the absorbing cross section is evaluated from the quality factors measurements [61,62,63,64], by using the relationship:

Q = 16 π^2^V<|S_21_|^2^>/λ^3^ η_Tx_ η_Rx_ (5)

where <|S_21_|^2^>, which is equal to <Pr>/<Pt> (where Pr is the power captured by the receiving antenna Rx with total radiation efficiency η_Rx_ = 1 − |<S_22_>|^2^ and Pt is the power injected by the transmitting antenna Tx with total radiation efficiency η_Tx_ = 1 − |<S_11_>|^2^), is related to the dielectric loss rate since S_21_ represents the coefficient of transmission between the two VNA ports. The relationships (2) to (5) are considered to be valid as long as the refraction index of the samples under test do not vary appreciably.

## 3. Results

### 3.1. Process Methodology

The novel concept introduced here aims to exploit the different capabilities of the tested materials in a synergic route. It is suggested that a hybrid ceramic/polymeric shell structure could be employed in order to ensure simultaneously extreme temperature withstanding and electromagnetic shielding behavior in two different spacecraft configurations: as the leading edge of a re-entry supersonic flight vehicle which aims to decrease its radar cross section (RCS) once back in Earth’s atmosphere, and as a thermal protection subsystem of a stealth cube satellite. One intriguing strategy to tackle such an issue has been picked out in the coupling between the C/C material and the carbon micro/nano-powder reinforced composite. Such an approach is due to the chemical affinity between the carbon matrix and carbon powder (basically made of the same element, i.e., graphitic carbon) being a likely route to solve the typical issues of thermo-mechanical compatibility.

**Figure 7:**
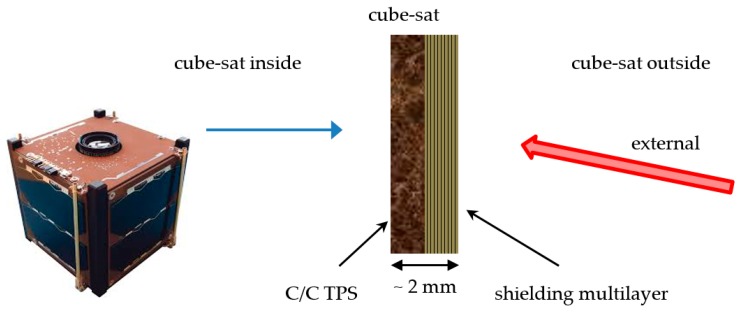
Sketch of the C/C multilayer hybrid structure in the stealth cube-sat configuration. TPS = thermal protection system.

**Figure 8:**
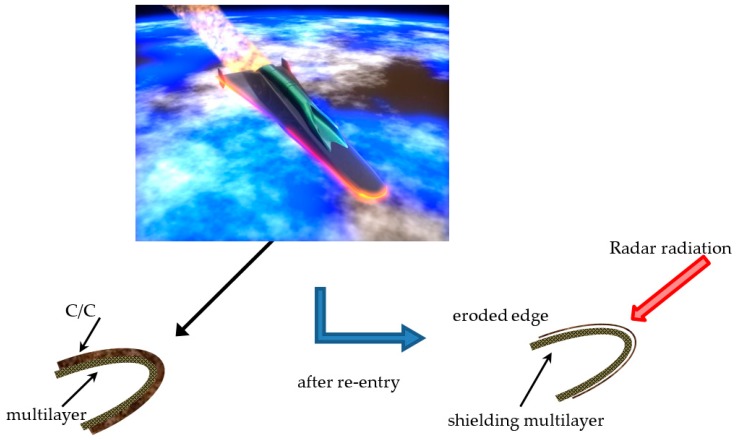
Sketch of the C/C multilayer hybrid structure in the stealth sub-orbital aircraft configuration.

In particular, the same coupling between a thin C/C stratification and a specific nanocomposite multilayer is proposed in the following assembly for two different multifunctional space applications:

- Tile for cube-sat faces—The external surface is the EM impinged layer of the optimized carbon nanocomposite multilayered plate-shaped structure, aimed at microwave shielding, i.e., the satellite’s radar cross section reduction; the inner ceramic C/C bulk is imposed as a thermo-structural component to preserve the integrity and the correct working of the internal instrumentation from the severe thermo-mechanical stress experienced by the satellite during in orbit operations (see Figure 7).
- b. Shell for leading edge of sub-orbital aircraft—The C/C coating is externally exposed to the space conditions as TPS, and it is dimensioned to be totally eroded during the critical re-entry phase; the underlying nanocomposite-based lamination then acts as stealth component during the orbital operations (see Figure 8).

The following sections will describe the experimental results obtained for the two components separately, then some numerical simulations will explore the potentiality of the multi-scale hierarchic carbon assembly for the proposed applications.

### 3.2. C/C Morphological and Thermo-Mechanical Analysis

The sample density was evaluated by the use of a Mettler-Toledo XP26DR (Columbus, OH, USA) balance with 2 μg sensitivity located in an ISO 8 clean room. Density is a very important parameter which allows us to evaluate the volatile part of the matrix. The specimens were weighed before and after the stabilization cycle; the results are reported in Table 3, where the total mass losses are also reported in terms of percentages. The mean value of the total mass loss is around 0.16%: that underlines how the matrix is well structured around the fibers and that there is a low content of volatile components, thus ensuring a good level of stability of the material. Moreover, such a result suggests that the improvement in the thermal and mechanical characteristics is due to a re-arrangement of the matrix morphology.

**Figure 9:**
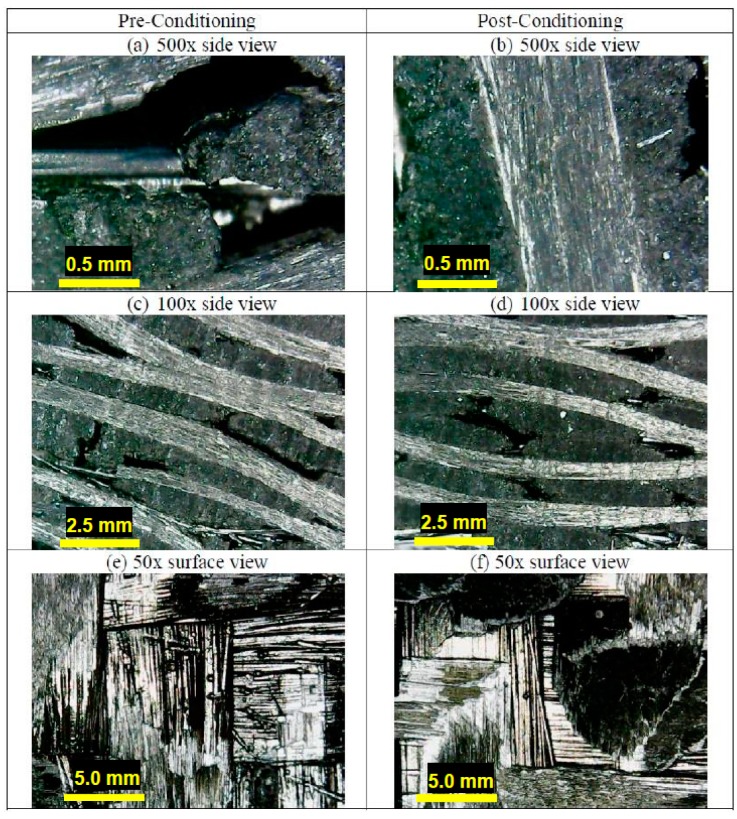
Optical images of the C/C samples.

A morphological analysis was carried out by means of a B-1000 Optika microscope (Bergamo, Italy). The principle of the confocal microscope is to illuminate only one spot on the sample through a pinhole: the light is reflected by the objective back to the pinhole, thus a complete image can be formed by scanning the spot of the sample in a raster pattern. The optical microscope images reported in Figure 9 show a clear improvement induced by the stabilization cycle on the material samples. In fact, the high magnification images highlight that the carbon matrix is much more attached to the carbon fibers after the treatment (Figure 9b) than what occurs before (Figure 9a). This is confirmed at medium magnification: although some voids are still visible, the interface appears more continuous in the treated material (Figure 9d) than in the pre-conditioning case (Figure 9c). The sample’s upper surface is shown by the low magnification images: the morphology of the C/C composite appears much neater after the cycle (Figure 9f) than what is visible for the untreated sample (Figure 9e). All these results can be explained by the creation of further matrix aggregates, thanks to the residual graphitic powder in the bulk of the samples: part of this powder was blown out during the vacuum pre-conditioning phase, but a residual content remained inside by aggregating at the matrix.

**Figure 10:**
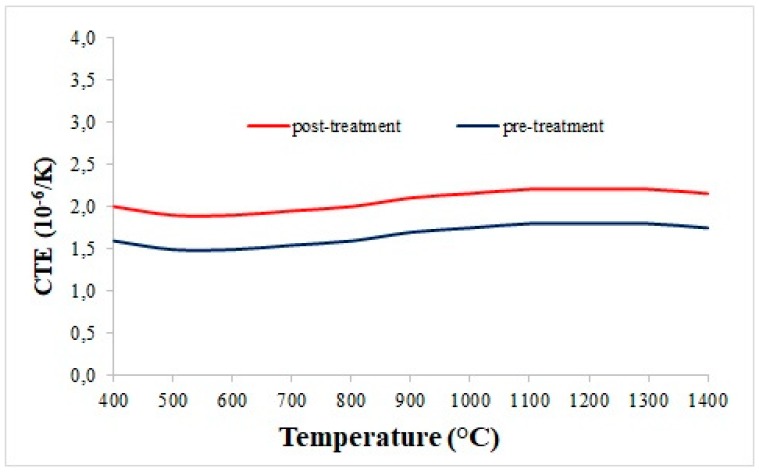
C/C thermal expansion coefficient before and after the stabilization cycle.

A thermal analysis of the C/C materials was performed by means of a push rod dilatometer (LINSEIS L75H, Selb, Germany). The test follows the ASTM E228 standard for the determination of the linear thermal expansion coefficient of rigid solid materials. The samples have been pre-conditioned in a humidity-controlled environment before the characterization. The control tests were performed on alumina samples in order to estimate the measurement accuracy, which resulted in 0.2 × 10^−6^/K. The dilatometric analysis was performed on both samples infiltrated by CVI as well as on samples which underwent the stabilization process. Table 4 reports the average values of the thermal expansion coefficients of pre-treatment and post-treatment samples. The mean percentage difference is around 9.3%: the corresponding value of about 0.17 × 10^−6^/K, standing within the range of measure accuracy, demonstrates that the thermal properties of the composites are stable with the increase in temperature. Accordingly, the CTE vs temperature plots show great thermal stability over 1000 °C, regardless of performing the stabilization cycle (Figure 10).

**Figure 11:**
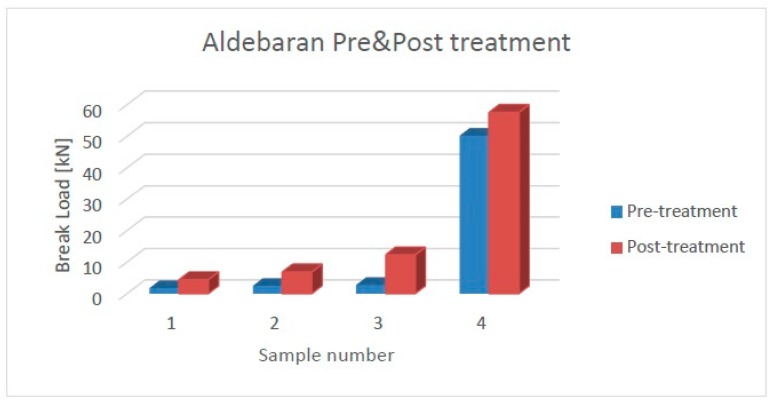
Aldebaran pre- and post-treatment samples compression strength.

**Figure 12:**
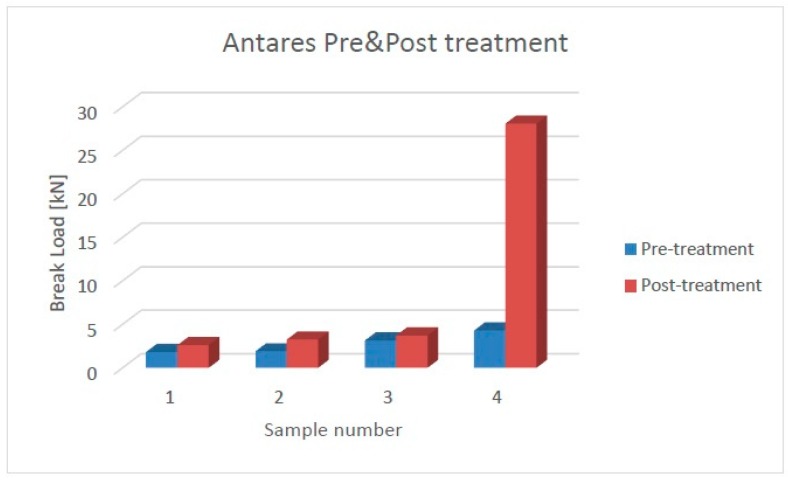
Antares pre- and post-treatment samples compression strength.

The mechanical analysis was performed by means of a Schenck Trebel (New York City, NY, USA) universal testing machine. The device was equipped with two force transducers—an U2B (class 00) 20 kN and a METIOR TRZ200 (Como, Italy) 2 kN, both with a nominal sensitivity 2 mV/V—and with two parallel plates in order to perform the compression test to assess the break load of the C/C composites. In the diagrams of Figure 11 and Figure 12, the results are reported for the specimens of the two infiltrated preforms, before and after the stabilization cycle, having the fibers partially oriented in a circumferential orientation (samples 1–3) and overall perpendicular with respect to the loading direction (sample 4). All the samples show an increase in the compression strength after the treatment: the mean improvement for Aldebaran is about 200%, while for Antares, it is about 40%. This trend is confirmed by evaluating the average values of the break load in the circumferential orientation, which were 2.3 ± 0.5 kN and 7.8 ± 3.7 kN for Aldebaran before and after the treatment, respectively, and 2.3 ± 0.7 kN and 3.2 ± 0.6 kN for Antares before and after the treatment, respectively. Such behavior is believed to be dependent on the matrix morphology variation, in agreement to what was observed by the morphological analysis.

### 3.3. Multilayers Absorbing Cross Section Evaluation

**Figure 13:**
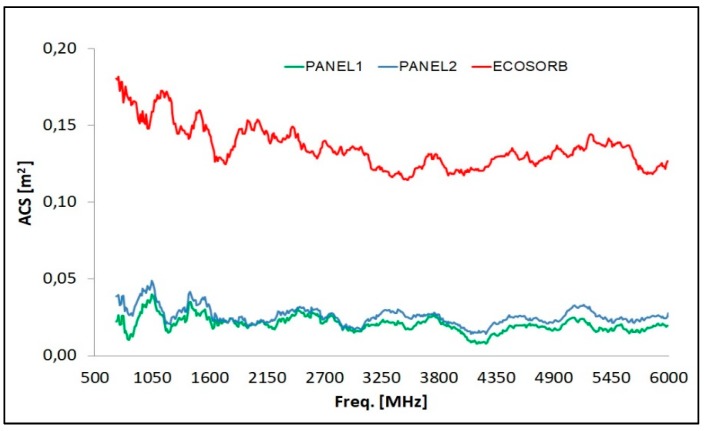
ACS of the micro- and nano-filled multilayered panels within the microwave S-band.

In Table 5, the S-band ACS of the two multilayered panels is reported: the values fall between 0.015 m^2^ and 0.035 m^2^, thus showing a non-absorbing behavior in the investigating microwave band, due to both the presence of graphitic material and to the multilayers impedance mismatch. The plot of the multilayers ACS vs frequency is given in Figure 13, together with the absorbing reference performance of an Ecosorb panel. As usual, the higher surface area provided by the carbon nanostructures (Panel 2) gives the final composites more effective properties (in this case, the reflection capability induced by an increase in electrical conductivity) comparable to those of the same matrix enriched with a much higher weight percentage of carbon micro-filler (Panel 1). Such results, together with the previously reported behavior around 12 GHz, suggest the possibility to use the nanocomposite multilayer for shielding in the X-band without compromising signals and communications in the S-band. A particular remark is necessary for Panel 2, as it shows an absorption peak around 3 GHz in free space (see Figure 4b), in apparent contrast with the result obtained in the RC environment. This is explained by considering the different mechanism of the characterization route. In the RC set-up, the microwaves surround the sample, thus interacting with all the layers of the panel, while in free space measurement, the incident waves only collide on the material’s surface, which in this case is made of epoxy resin: this resin is able to provide the impedance matching condition, which represents a fundamental requirement for EM absorption.

### 3.4. Numerical Simulation of Hybrid TPS/Stealth Structures

A finite element method (FEM) analysis has been performed by the ANSYS (Canonsburg, PA, USA) simulation tool in order to gain a qualitative hint about the thermal behavior of the proposed hybrid structure in the two operating configurations introduced above.

**Figure 14:**
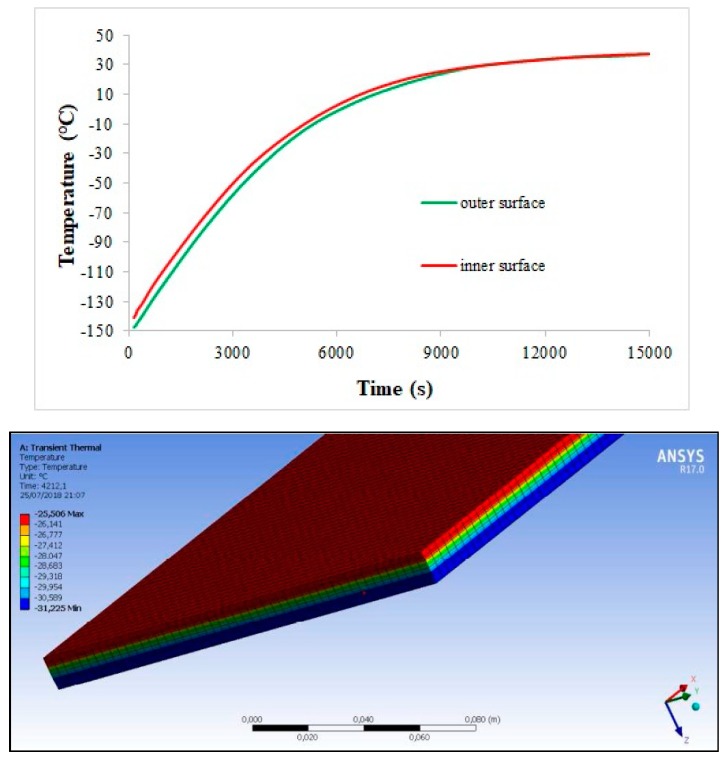
Finite element method (FEM) simulation: temperature variation in the hot phase, during the thermal cycle (**up**) and along the structure thickness at fixed time (**down**).

**Figure 15:**
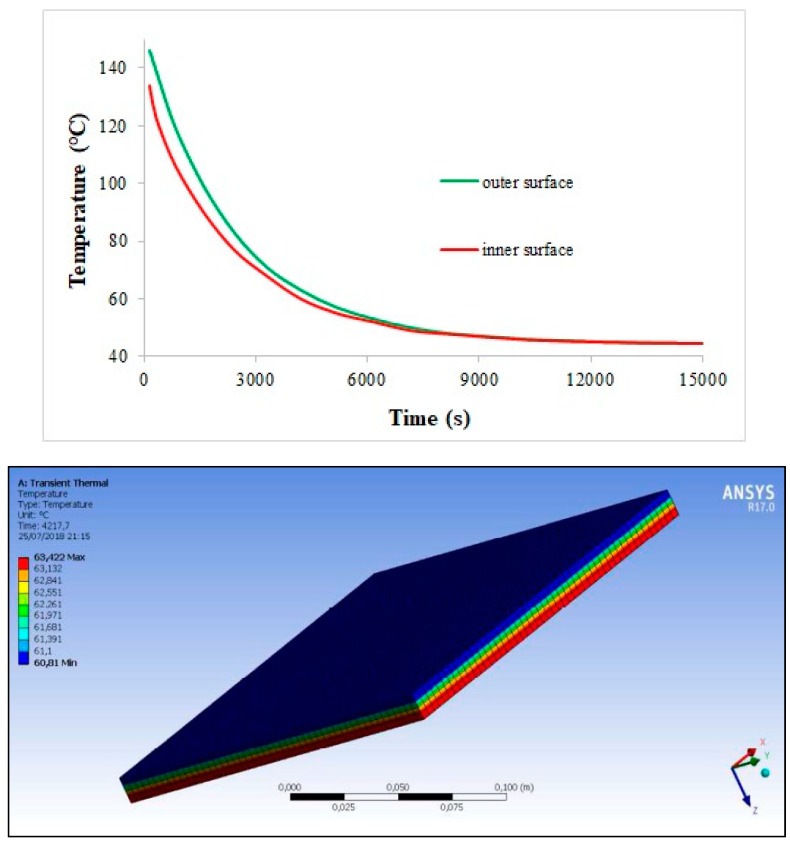
FEM simulation: temperature variation in the cold phase, during the thermal cycle (**up**) and along the structure thickness at fixed time (**down**).

As far as the first application is concerned (see Figure 7), the temperature variation along the multilayer–C/C assembly during a typical space thermal cycle is evaluated. The thermal flux is considered constant during the two phases of a single cycle and is fixed to 260 W/m^2^ and 225 W/m^2^ with initial temperature conditions of −150 °C and +150 °C during the hot and cold step, respectively. The total thickness of the multilayer is 9.4 mm (with layer thickness as in Figure 3b), while the C/C tile is designed to be 3.0 mm thick. The density of C/C is fixed to 1.50 g/cm^3^, whereas the three nanocomposite layers have densities around 1.15 g/cm^3^ (as well known, the MWCNT inclusion does not affect the matrix density, due to the high nanoparticle surface area). Within the explored temperature range, thermal conductivity and specific heat of the multilayer are supposed constant and fixed respectively to 0.15 W/mK and 1000 J/kg °C for the naked layer, to 1.00 W/mK and 1300 J/kg °C for the 1.0 wt% reinforced layer, and to 1.50 W/mK and 1400 J/kg °C for the 1.5 wt% reinforced layer. C/C thermal conductivity and specific heat fall in the ranges 2.9–3.8 W/mK and 420–1070 J/kg °C from cold to hot temperature, respectively. Figure 14 and Figure 15 report the temperature variation during the cycle (at the outer and inner surface (green and red curve of the plot, respectively)) and along the assembly thickness (at a fixed instant) for the hot and cold phase. In both the cases, the temperature reaches an asymptotic value after 250 min approximately, just below and above 40 °C in the hot and cold phase, respectively. In this framework, it can be argued that the C/C thin slab is able to ensure good thermal protection of the spacecraft inside; in absence of the high thermal conductivity region, in fact, it is believed that the temperature would be critically increased during the hot step, as indicated by the low temperature differences induced by the nanocomposite layers.

**Figure 16:**
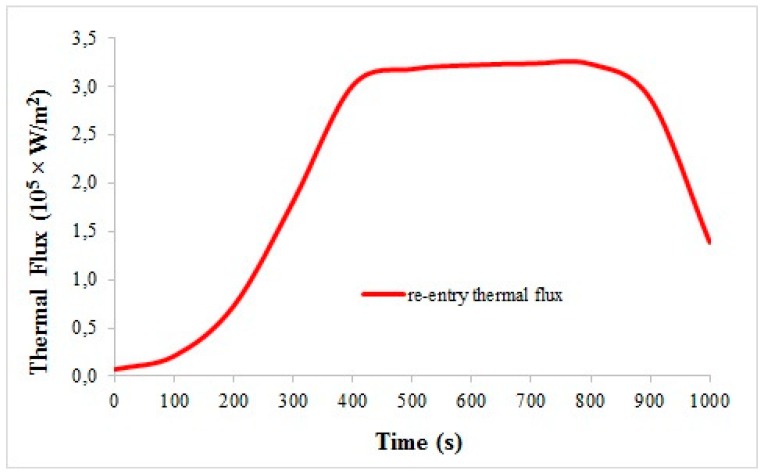
Thermal flux experienced by spacecraft during re-entry in earth atmosphere.

**Figure 17:**
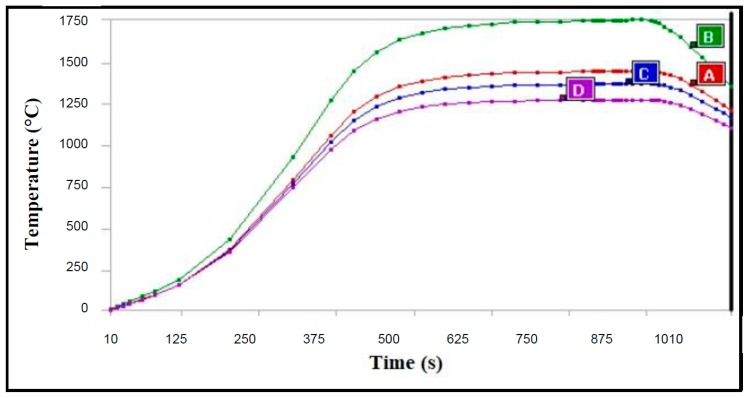
Temperature profile of a 1 cm thick C/C tile during re-entry phase. The profile is reported at four fixed heights of the C/C layer, from the upper exposed surface (probe B) down to the substrate (probe D), probes A and C representing intermediate points.

As far as the second application is concerned (see Figure 8), the critical re-entry conditions are simulated over a 1 cm thick C/C tile in order to evaluate the thermal protection level available for the stealth substrate. In Figure 16, the adopted thermal flux is plotted against time. Within the experienced thermal range, C/C thermal conductivity and specific heat vary in the ranges of 2.9–6.3 W/mK and 420–2040 J/kg °C (toward higher temperatures), respectively. Figure 17 reports the temperature variation during the re-entry phase along the TPS structure, i.e., in four fixed points, from the upper exposed surface (probe B) down to the substrate (probe D). Having established that the C/C structure provides impressive thermal control against the severe thermal flux of the re-entry environment, it is recognized that long before the highest thermal flux phase, the composite multilayer underneath would suffer from too harsh thermal conditions (several hundreds of °C).

## 4. Conclusions

In this paper, the authors have presented a synthesis of part of the experimental activity research carried out on advanced composite materials for space applications, focusing on specific aerospace solutions by means of multi-functional carbon-based composite assembly.

A technical objective to gain by using engineered materials for space activities is providing two or more specific functionalities by means of single items/components. In such a framework, carbon-based composites are believed to be key materials within the forthcoming drawing up and development of deep space missions, allowing us to realize a variety of multi-functional structures for different purposes.

Thermal protection from the aggressive space environment is one of the main tasks to tackle in space vehicle design and construction. Carbon-based ceramic composites are the most promising materials for such a kind of application, due to their impressive thermal stability and lightweight structure. The manufacturing of high thickness C/C prototypes for re-entry systems by means of a CVI process was presented, and the thermo-mechanical analysis of the C/C shell-shaped specimens was reported. It has been shown that an enhanced thermal treatment may improve the matrix microstructural geometry and thus the thermo-mechanical characteristics of the densified materials. Such a stabilization process at low temperatures was analyzed in order to improve the final performance of the produced C/C material with the aim to provide a more cost-effective solution compared to expensive graphitization processes. The proposed treatment, performed only for 90 min at 400 °C, showed a significant upgrade of the composite mechanical properties, thanks to the increase of the matrix bonding to the fibers, whereas the thermal behavior remained stable before and after the stabilization cycle.

With the aim to conceive a compact structure able to provide thermal protection and low radar observability simultaneously, microwave absorbing cross section measurements of RAM space components made of micro-/nano-carbon reinforced polymeric composites were reported. The microwave absorption efficacy of specifically designed multilayers was established. Further studies will be focused on the production of plates based on genetic algorithms, in order to optimize the thickness (e.g., the weight of the panel) and the composition of the different layers. Moreover, reflection and transmission measurements will be carried out on these plates in order to better characterize the material behavior over microwave broad bands.

Finally, the possibility of combining the C/C bulk with a carbon-reinforced skin in a synergic arrangement was discussed, with the aid of numerical and experimental analyses. The research described here suggests that a hybrid ceramic/polymeric shell structure could be effectively employed as a spacecraft specific subsystem in order to ensure simultaneously extreme temperature withstanding and EM shielding behavior in two different spacecraft configurations. As far as the thermal protection structure of a stealth cube satellite is concerned, the simulated behavior highlighted the effective properties provided by the proposed solution; in fact, the upper multilayered laminate ensures the required microwave absorption, while the bottom C/C thin slab is able to safeguard the spacecraft inside by providing suitable thermal protection. Regarding the application as the leading edge of a re-entry supersonic flight with decreased RCS once back in Earth’s atmosphere, despite the thermal protection provided by the C/C component, the underneath composite would be seriously affected by the harsh thermal flux conditions; further analyses will be performed to envisage the feasibility of this kind of application, including the possibility of isolating the inner multilayer by means of a thermal protective coating made of EM wave non-interacting material.

## Figures and Tables

**Table 1:** Mechanical properties of a typical unidirectional carbon/carbon (C/C) composite at various temperatures [39].

Property	Temperature, °F (°C)			
	70 (21)	1500 (816)	3000 (1649)	3500 (1927)
Tensile strength-warp longitudinal direction, psi (MPa)	48.0 × 10^3^ (331)	54.0 × 10^3^ (372)	60.0 × 10^3^ (414)	62.0 × 10^3^ (427)
Tensile strength-fill transverse direction, psi (MPa)	40.0 × 10^3^ (276)	45.0 × 10^3^ (310)	50.0 × 10^3^ (345)	–
Modulus-warp longitudinal direction, psi (GPa)	16.5 × 10^6^ (114)	16.5 × 10^6^ (114)	15.0 × 10^6^ (103)	12.5 × 10^6^ (86)
Modulus-fill transverse direction, psi (GPa)	15.8 × 10^6^ (109)	15.8 × 10^6^ (109)	14.4 × 10^6^ (99)	12.0 × 10^6^ (83)
Compressive strength-warp longitudinal direction, psi (MPa)	26.0 × 10^3^ (179)	33.0 × 10^3^ (228)	40.0 × 10^3^ (276)	–
Compressive strength-fill transverse direction, psi (MPa)	26.0 × 10^3^ (179)	30.0 × 10^3^ (207)	35.0 × 10^3^ (241)	–

**Table 2:** Main characteristics of the two manufactured C/C prototypes.

Item	Fiber Yarn & D-Stitch	Thickness (mm)	Density (g/cm^3^)
Antares	12 K/3D	30–36	1.52
Aldebaran	12 K/4D	30–36	1.46

**Table 3:** Material samples weight and mass loss during the stabilization cycle.

Item	Sample Number	Weight before Cycle (g)	Weight after Cycle (g)	Total Mass Loss (%)
Antares	1	37.30	37.25	0.13
	2	36.51	36.44	0.19
	3	36.70	36.65	0.14
	4	37.56	37.51	0.13
Aldebaran	1	35.00	34.94	0.17
	2	26.45	26.40	0.19
	3	34.65	34.61	0.12
	4	38.67	38.60	0.18

**Table 4:** The coefficient of thermal expansion (CTE) measurement results for C/C samples.

Temperature (°C)	Average Value of Pre-Treatment (10^−6^/K)	Average Value of Post-Treatment (10^−6^/K)	Difference (%)
400	1.60	1.60	0
500	1.60	1.60	0
600	1.60	1.70	6.25
700	1.60	1.70	6.25
800	1.70	1.80	5.88
900	1.70	1.90	11.76
1000	1.70	2.00	17.65
1100	1.80	2.00	11.11
1200	1.90	2.10	10.53
1300	1.90	2.10	10.53
1400	1.90	2.20	15.79
1500	1.90	2.20	15.79

**Table 5:** Absorbing cross section (ACS) values of the multilayered panel in the S-band.

Frequency (MHz)	ACS-Panel 1 (m^2^)	ACS-Panel 2 (m^2^)
2200	0.020	0.020
2250	0.025	0.030
2500	0.027	0.032
2750	0.025	0.026
3000	0.017	0.019
3250	0.021	0.031
3500	0.022	0.026
3750	0.026	0.027
4000	0.017	0.022
